# Phase I/II trial evaluating carbon ion radiotherapy for the treatment of recurrent rectal cancer: the PANDORA-01 trial

**DOI:** 10.1186/1471-2407-12-137

**Published:** 2012-04-03

**Authors:** Stephanie E Combs, Meinhard Kieser, Daniel Habermehl, Jürgen Weitz, Dirk Jäger, Piero Fossati, Roberto Orrechia, Rita Engenhart-Cabillic, Richard Pötter, Manjit Dosanjh, Oliver Jäkel, Markus W Büchler, Jürgen Debus

**Affiliations:** 1 Deparment of Radiation Oncology, Im Neuenheimer Feld 400, University Hospital of Heidelberg, Heidelberg 69120, Germany; 2 Department of Biostatistics, Im Neuenheimer Feld 305, University Hospital of Heidelberg, Heidelberg 69120, Germany; 3 Department of Surgery, University Hospital of Heidelberg, Im Neuenheimer Feld 110, Heidelberg 69120, Germany; 4 Nationales Centrum für Tumorerkrankungen (NCT), Medizinische Onkologie, Im Neuenheimer Feld 360, Heidelberg 69120, Germany; 5 Fondazione CNAO - Centro Nazionale di Adroterapia Oncologica, Via Caminadella, 16, Milano 20123, Italy; 6 Department of Radiation Oncology, Baldingerstraße, University Hospital of Marburg, Marburg 35043, Germany; 7 Department of Radiation Oncology, University of Vienna, Währinger Gürtel 18-20, Wien 1090, Austria; 8 CERN, Geneva 23 1211, Switzerland; 9 Heidelberger Ionenstrahl Therapiezentrum (HIT), Im Neuenheimer Feld 450, Heidelberg 69120, Germany

## Abstract

**Background:**

Treatment standard for patients with rectal cancer depends on the initial staging and includes surgical resection, radiotherapy as well as chemotherapy. For stage II and III tumors, radiochemotherapy should be performed in addition to surgery, preferentially as preoperative radiochemotherapy or as short-course hypofractionated radiation. Advances in surgical approaches, especially the establishment of the total mesorectal excision (TME) in combination with sophisticated radiation and chemotherapy have reduced local recurrence rates to only few percent. However, due to the high incidence of rectal cancer, still a high absolute number of patients present with recurrent rectal carcinomas, and effective treatment is therefore needed.

Carbon ions offer physical and biological advantages. Due to their inverted dose profile and the high local dose deposition within the Bragg peak precise dose application and sparing of normal tissue is possible. Moreover, in comparison to photons, carbon ions offer an increase relative biological effectiveness (RBE), which can be calculated between 2 and 5 depending on the cell line as well as the endpoint analyzed.

Japanese data on the treatment of patients with recurrent rectal cancer previously not treated with radiation therapy have shown local control rates of carbon ion treatment superior to those of surgery. Therefore, this treatment concept should also be evaluated for recurrences after radiotherapy, when dose application using conventional photons is limited. Moreover, these patients are likely to benefit from the enhanced biological efficacy of carbon ions.

**Methods and design:**

In the current Phase I/II-PANDORA-01-Study the recommended dose of carbon ion radiotherapy for recurrent rectal cancer will be determined in the Phase I part, and feasibilty and progression-free survival will be assessed in the Phase II part of the study.

Within the Phase I part, increasing doses from 12 × 3 Gy E to 18 × 3 Gy E will be applied.

The primary endpoint in the Phase I part is toxicity, the primary endpoint in the Phase II part is progression-free survival.

**Discussion:**

With conventional photon irradiation treatment of recurrent rectal cancer is limited, and the clinical effect is only moderate. With carbon ions, an improved outcome can be expected due to the physical and biological characteristics of the carbon ion beam. However, the optimal dose applicable in this clincial situation as re-irradiation still has to be determined. This, as well as efficacy, is to be evaluated in the present Phase I/II trial.

**Trial registration:**

NCT01528683

## Background

Treatment of rectal cancer after primary diagnosis depends on initial staging and includes surgical resection, radiation therapy as well as chemotherapy. For T1-2 tumors without positive lymph nodes, surgical resection alone followed by close oncological follow-up is recommended. For node-negative T3 tumors, surgery should be followed by radiochemotherapy followed by adjuvant chemotherapy. In patients with node positive T1-3 tumors, surgical resection should also be followed by radiochemotherapy and adjuvant chemotherapy [1]. There is substantial evidence that radiation therapy prior to surgical resection is beneficial with respect to outcome. For example, pre-operative radiochemotherapy has been shown to be superior to postoperative radiotherapy in stage II-III tumors reducing local failure rates from 13% to 6% in the preoperative arm [2,3]. It should be followed by surgical resection and adjuvant chemotherapy. In this concept, toxicity is substantially lower than with postoperative radiochemotherapy, and significant downstaging can be achieved with radiochemotherapy prior to surgery. In the preoperative concept as well as in the postoperative radiation treatment, generally, doses of 45 Gy with a boost of 5.4 - 9 Gy to the macroscopic tumor or tumor bed are applied. Short course radiotherapy with 5x5 Gy is another alternative which can be performed prior to surgical resection, and has shown significant reduction of local recurrences compared to surgery alone [4-10].

Advances in surgical techniques such as the establishment of the total mesorectal excision (TME) in combination with advanced radiation and chemotherapy have reduced local failure rates to few percent only [8,11,12]. However, recurrences do occur, and treatment options at this stage can be limited due to the size and location of the lesion, as well as due to previously performed treatments including radiation therapy.

Surgical resection should be evaluated in all instances, and can be a treatment option in this situation for certain patients [13]. However, in some cases a resection is not possible, or medical reseasons such as concomitant illnesses restrain the surgeon from surgical interventions. In other cases, surgery is performed, but a gross resection is not possible and macroscopic tumor remains which requires adjuvant treatment.

With advanced photon techniques delivering doses precisely through three-dimensional CT- and MR-based treatment planning, re-irradiation can be performed for recurrent rectal cancer, however, doses are commonly limited to 36 - 45 Gy applied with small safety margins due to the normal tissue exposure during prior radiotherapy. In the past, neutrons had been used for the treatment of recurrent rectal cancer; clinical results with respect to pain control and and local progression-free survival were between 50 and 85% in the different centers [14,15].

Particle therapy using protons or carbon ions offers distinct physical and biological properties compared to photon radiotherapy. The physical characteristics include a low dose deposition within the entry channel of the particle beam, followed by a steep dose deposition called the Bragg Peak, which is followed by a sharp dose fall-off. Additionally carbon ions offer significant biological advantages through severe radiation damage performed within the irradiated cells which are difficult to repair by the cells' intrinsic repair mechanisms. For various cell lines, RBE values between 2 and 5 have been reported depending on the cell line and endpoint. Therefore, carbon ion radiotherapy is characterised by a higher relative biological effectiveness (RBE) which can translate into improved clinical results.

Carbon ion radiotherapy was available by the Department of Radiation Oncology at the Gesellschaft für Schwerionenforschung (GSI) in Darmstadt since 1997. Superior treatment results for a number of tumor entities, such as chordomas and chondrosarcomas of the skull base, as well as adenoid cystic carcinomas (ACC) have been shown, and carbon ion radiotherapy is currently performed in the clinical routine for these patients [16-19]. Safety of carbon ion radiotherapy with respect to critical organs at risk, such as the brain, brainstem or spinal chord, have been shown in these studies. At the Heidelberg Ion Therapy Center (HIT), treatment of over 1300 patients per year with proton and carbon ion RT is possible.

In Japan, carbon ion treatment has been available for over 15 years, and over 5000 patients with different indications have been treated successfully showing excellent clinical results [20,21]. For recurrent rectal cancer, excellent local control rates significantly higher than those obtained after surgical resection have been achieved with carbon ions; mostly, these patients were all initially diagnosed with stage I tumors not treated with radiation and/or chemotherapy after primary diagnosis.

Due to the beneficial dose distributions generated by the particle beam and the higher RBE with carbon ions, the use of carbon ion radiotherapy for recurrent rectal carcinoma is a promising treatment alternative in this patient population. To date, few reports have focussed on re-irradiation using percutaneous photons or intraoperative radiotherapy with electrons in patients with recurrent rectal cancer.

In general, recurrent rectal cancers are treatment-resistant tumors, and local high-dose radiation treatment is often limited by organs at risk as well as previously applied radiotherapy after initial diagnosis. Japanese studies using carbon ion radiotherapy for the treatment of recurrences from rectal cancer previously not treated with radiation have shown excellent local control rates which are superior to conventional radiation therapy as well as surgery alone.

In a first step, a dose-escalation study had been performed from 67.2 Gy E to 73.6 Gy E in 16 fractions. Local control rates were 93.7% at 5 years at the dose level of 73.6 Gy E with very low rates of treatment related acute or chronic side effects [20,21]. However, these patients had not been treated with prior radiotherapy, due to the tumor stage they had only been treated with surgical resection after primary diagnosis.

Therefore, the concept of carbon ion radiotherapy to patients with recurrent rectal cancer with a macroscopic tumor lesion after aggressive primary treatment including radiotherapy is a promising treatment alternative. Due to the physical properties of the particle beam, sparing of normal surrounding tissue supports the use of this concept as re-irradiation in patients with recurrent rectal cancer.

Therefore, in the PANDORA-01-Study, this concept will be evaluated; in the first step, the recommended dose will be determined in a dose escalation scheme (Phase I part) prior to the Phase II part of the study.

Due to the dose escalation part within this prospective study, the safety of a recommended dose (RD) of carbon ion radiotherapy will be determined using a classical 3 + 3 design. Within the Phase II part, the RD outcome after carbon ion radiotherapy will be evaluated. Results will then be compared to historical controls treated with surgery alone or with conventional radiation techniques or Intraoperative Electron Radiotherapy (IOERT). Patients will be monitored regularly to observe and evaluate treatment outcome and side effects.

At the University of Heidelberg, patients with rectal cancer are treated in the interdisciplinary setting consisting of visceral surgeons, medical oncologist and radiation oncologist. Therefore, patients will be provided the best possible oncological care on a professional basis.

## Methods and design

The purpose of the trial is to determine the MTD for carbon ion radiotherapy for the treatment of recurrent rectal cancer and to determine feasibility of this treatment in patients with recurrent rectal cancer.

### Primary objective

#### Phase I

The primary endpoint is any Grade IV toxicity related to the study treatment according to CTCAE Grade 4.0.

#### Phase II

The primary endpoint is progression-free survival after re-irradiation at 12 months.

### Secondary objectives

#### Phase I

The secondary endpoint in the Phase I part is progression-free survival after re-irradiation

#### Phase II

The secondary endpoints in the Phase II part are overall survival, toxicity and safety.

### Trial design

The trial will be performed as a single-center one-armed Phase I/II study.

#### Phase I: Dose escalation

Patients fulfilling the inclusion criteria will be treated with increasing total doses of carbon ion radiotherapy to evaluate the optimal carbon ion dose with respect to toxicity. The aim of this part is to determine the MTD of carbon ion radiotherapy for re-irradiation of recurrent rectal cancer.

Patients will be treated within seven increasing dose regimens starting at 12 × 3 GyE up to 18 × 3 GyE.

#### Phase II: Treatment with RD

Patients fulfilling the inclusion criteria will be included into the Phase II part of the study and treated with the MTD determinated in the Phase I part.

### Trial duration and schedule

#### Phase I dose escalation part

The primary endpoint is toxicity measured by any Grade IV toxicity related to the study treatment according to CTCAE Grade 4.0. A maximum of 45 patients are projected for the Phase I part of the study. Patients will be followed for at least 3 months after study treatment to document any toxicity according to CTCAE Version 4.0.

#### Phase II part

The primary endpoint is progression-free survival after re-irradiation at 12 months, therefore patients are followed within the trial protocol for a minimum 12 months after completion of study treatment. For the LPI, the final study visit will be 12 months after study treatment to assess the primary endpoint. All other patients will be followed on a regular basis as stated below until death or until 12 months after LPI.

After RT, patients are scheduled for follow-up visits every 2 months or as needed clinically including contrast-enhanced MRI or CT, thorough clinical assessment as well as evaluation of blood values including CEA.

The last patient included into the study will be followed 12 months after treatment. This is considered the final study visit. All other patients will be followed regularly as described in detail until death or until 12 months after LPI.

The overall duration of the trial is expected to be approximately 36 months. Recruitment of the patients is planned over a time period of 24 months, minimum duration of the follow-up phase will be 12 months.

### Patient selection: Inclusion and exclusion criteria

#### General criteria for patients' selection

Patients with the diagnosis of recurrent rectal cancer will be evaluated and screened for the protocol. All patients fulfilling the inclusion and exclusion criteria will be informed about the study. Patients will be included according to the incidental gender distribution for patients with recurrent rectal cancer, male and female patients will be included.

#### Inclusion criteria

Patients meeting all of the following criteria will be considered for admission to the trial:

- Locally recurrent rectal cancer

- Inoperable lesion

- Macroscopic tumor up to 1000 ml volume

- Prior photon irradiation of 20-60 Gy

- Time between initial radiotherapy and re-irradiation of at least 12 months

- Age ≥ 18 years of age

- Karnofsky Performance Score ≥60

- For women with childbearing potential, (and men) adequate contraception.

- Ability of subject to understand character and individual consequences of the clinical trial

- Written informed consent (must be available before enrolment in the trial)

### Exclusion criteria

Patients presenting with any of the following criteria will not be included in the trial:

- Refusal of the patients to take part in the study

- Advanced metastatic disease

- Patients who have not yet recovered from acute toxicities of prior therapies.

- Known carcinoma < 5 years ago (excluding Carcinoma in situ of the cervix, basal cell carcinoma, squamous cell carcinoma of the skin) requiring immediate treatment interfering with study therapy.

- Pregnant or lactating women

- Participation in another clinical study or observation period of competing trials, respectively.

### Prior and concomitant treatments

During radiation therapy, the application of steroids or other supportive medication might be necessary due to the nature of the disease. Addtionally, medication required for the individual patient for the underlying illness or for concomitant illnesses (i.e. hypertension, thyroid disease, hyperlipidemia etc.) can be applied. Concomitant medication should be discussed with the principal investigator on an individual basis.

No concomitant chemotherapy or any other anti-tumor medication is allowed during the treatment period in this trial.

### Radiation therapy

#### Treatment planning

For particle therapy, patients will be immobilized using an individual fixation system. For treatment planning, contrast-enhanced CT as well as MR-imaging will be performed for optimal target definition.

Patients included to the study will have received 20-60 Gy of photon RT.

Organs at risk such as the small intestine, bladder, spinal chord and cauda will be contoured. Dose constraints of normal tissue will be respected according to Emami et al. [22]. The Gross Tumor Volume (GTV) will be defined for the carbon ion treatment as the area of contrast enhancement on T1-weighted MR-imaging; the Clinical Target Volume (CTV) will be defined as the GTV adding a safety margin of 5-10 mm depending on the clinical situation and the location of the lesion to account for potential microscopic spread.

A planning target volume (PTV) will be added depending on individual factors such as patient positioning or beam angles chosen and will be between 3 and 10 mm.

FDG-PET or SPECT-Examinations may be used in addition to contrast-enhanced MRI for target volume definition but are not mandatory.

Carbon ion RT planning is performed using the treatment planning software PT-Planning (Siemens, Erlangen, Germany) including biologic plan optimization. Biologically effective dose distributions will be calculated using the a/ß ratio for rectal cancer as well as for the endpoint late toxicity.

No interruptions > 4 days are allowed.

Patient positioning prior to particle therapy will be evaluated by comparison of x-rays to the DRRs. Set up deviations > 3 mm are corrected prior to radiotherapy.

### Dose prescription experimental (carbon) arm

The intensity-controlled rasterscan system will be used for beam application. Single fractions of 3 Gy E will be applied up to the total dose in the dose escalation cascade.

Seven dose levels are planned within the Phase I part:

12 × 3 Gy E

13 × 3 Gy E

14 × 3 Gy E

15 × 3 Gy E

16 × 3 Gy E

17 × 3 Gy E

18 × 3 Gy E

The dose will be prescribed to the maximum of the calculated dose distribution for the target volume (PTV). Treatment planning aims in the coverage of the PTV by the 90%-isodose line.

Dose specification is based on biologic equivalent dose because of the high relative biologic effectiveness (RBE) of carbon ions, which differs throughout the target volume due to its dependence on various factors. RBE will be calculated at each voxel throughout the target volumes and biological optimization will be performed. The dose prescription used is related to the isoeffective dose GyE (Gray equivalent) using daily fractions of 2 Gy and a weekly fractionation of 5 × 2 Gy.

After the RD has been determined, this dose will be the prescribed dose in the Phase II part of the study.

### Treatment assignment

Radiation therapy according to the protocol will be performed in patients included into the study.

Patients withdrawn from the trial retain their identification codes. New patients must always be allotted a new identification code.

### Assessment of efficacy parameters

#### Progression-free survival

Efficacy of the treatment will be recorded according to the RECIST Criteria.

#### Baseline documentation of "target" lesion

• The main target lesion is defined as the macroscopic tumor delineated for treatment with carbon ion radiotherapy.

• A sum of the longest diameter (LD) for the target lesions will be calculated and reported as the baseline sum LD. The baseline sum LD will be used as reference by which to characterize the objective tumor.

• All other lesions (or sites of disease) should be identified as non-target lesions and should also be recorded at baseline. Measurements of these lesions are not required, but the presence or absence of each should be noted throughout follow-up.

### Evaluation of the target lesion

• *Complete Response (CR)*: Disappearance of the target lesions

• *Partial Response (PR)*: At least a 30% decrease in the sum of the LD of the target lesion, taking as reference the baseline sum LD

• *Stable Disease (SD)*: Neither sufficient shrinkage to qualify for PR nor sufficient increase to qualify for PD, taking as reference the smallest sum LD since the treatment started

• *Progressive Disease (PD)*: At least a 20% increase in the sum of the LD of the target lesion, taking as reference the smallest sum LD recorded since the treatment started or the appearance of one or more new lesions

### Evaluation of non-target lesions

• *Complete Response (CR)*: Disappearance of all non-target lesions and normalization of tumor marker level

• *Incomplete Response/Stable Disease (SD)*: Persistence of one or more non-target lesion(s) or/and maintenance of tumor marker level above the normal limits

• *Progressive Disease (PD): *Appearance of one or more new lesions and/or unequivocal progression of existing non-target lesions

### Evaluation of best overall response

The best overall response is the best response recorded from the start of the treatment until disease progression/recurrence (taking as reference for PD the smallest measurements recorded since the treatment started). In general, the patient's best response assignment will depend on the achievement of both measurement and confirmation criteria

• Patients with a global deterioration of health status requiring discontinuation of treatment without objective evidence of disease progression at that time should be classified as having "symptomatic deterioration". Every effort should be made to document the objective progression even after discontinuation of treatment.

• In some circumstances it may be difficult to distinguish residual disease from normal tissue. When the evaluation of complete response depends on this determination, it is recommended that the residual lesion be investigated (fine needle aspirate/biopsy) to confirm the complete response status.

### Survival

Survival is a secondary endpoint of the study. The duration of survival is the time interval between beginning of carbon ion radiotherapy and the dated of death due to any cause. Patients not reported dead or lost to follow-up will be censored at the date of the last follow-up examination.

### Assessment of safety parameters

This study will use the International Common Terminology Criteria for Adverse Events (CTCAE) version 4.0 for toxicity and adverse event reporting. A copy or the CTCAE can be accessed from the CTEP home page.

Safety and toxicity of the study treatment will be evaluated by clinical examination as well as imaging studies (MRI or CT).

### Assessment of further parameters

The following parameters will be collected and taken into account in: CEA, age, Karnofsky Performance Score, lesion size.

### Plan for treatment or care after the trial

After completion of study treatment, no further treatment is planned and patients wtill be followed up regularly. Follow-up examinations include clinical assessment, contrast-enhanced imaging with MRI or CT, and evaluation of blood values including CEA. Patients will be seen every 2 months after treatment or as needed clinically.

Any systemic treatment or chemotherapy is not part of the clinical trial.

For tumor progression, treatment alternatives will be evaluated and discussed interdisciplinary considering options of surgical resection, systemic treatment such as chemotherapy, a third course of radiation therapy, or other.

### Statistical considerations

This section describes the considerations underlying the choice of the sample size as well as the statistical methodology applied for the analysis of the Phase I and the Phase II part of the PANDORA-01 study. By combining the Phase I and Phase II part within a single study, the results of those 9 patients that received the MTD within the Phase I part can be used for the assessment of the efficacy in the Phase II part. Therefore, the required sample size of the Phase II part can be reduced, which is highly desirable from an ethical point of view. More details on the evaluation can be found in the statistical analysis plans prepared for the two study parts which will be finalized prior to performing any analyses and which have to be authorized by the study biostatistician and the principal investigator.

### Phase I part of PANDORA-01

It is the aim of the Phase I part of this study to determine the MTD for carbon ion radiotherapy for the treatment of recurrent rectal cancer. The primary endpoint is the occurrence of a dose limiting toxicity defined as any Grade IV toxicity according to CTCAE Version 4.0, possibly, probably or definitely associated to study treatment and occurring during 30 days after completion of the study treatment.

The calculation of the sample size for the Phase I part of the PANDORA-01 trial is based on the traditional 3 + 3 dose escalation scheme which is conducted as follows:

Patients are treated in cohorts of three each receiving the same dose. For the assessment of a dose limiting toxicity (see definition above) patients are observed for 30 days after application of the study treatment.

If none of the three patients of a cohort exhibits a dose limiting toxicity, the next cohort of three patients receives the next higher dose.

Otherwise, if at least one patient of a cohort exhibits a dose limiting toxicity, a further cohort of three patients is treated at the same dose level without escalating the dose.

If exactly one out of the six patients treated at this dose exhibits a dose limiting toxicity, the trial continues as planned at the next higher dose level.

If two or more patients out of the six patients treated at this dose exhibit a dose limiting toxicity, the dose escalation stops at that level and the next lower dose is considered as the MTD. When the escalation has stopped, additional patients will be treated at the MTD to a total of nine patients.

The Phase I part of the trial is conducted to determine the MTD of carbon ion radiotherapy by consideration of a total of seven dose levels. Therefore, the maximum sample size is 45 patients (six dose levels with a maximum of 6 patients each and 9 patients at the MTD).

Primary endpoint to determine the MTD that is chosen out of seven dose levels is any Grade IV toxicity according to CTCAE Version 4.0, possibly, probably or definitely associated to study treatment and occurring during 30 days after completion of the study treatment. Secondary endpoints are other safety data on the applied dose levels as well as response, progression-free survival, and overall survival.

### Phase II part of PANDORA-01

The primary objective of the Phase II part of PANDORA-01 is to evaluate the 12 month progression-free survival rate *π *for patients with recurrent rectal cancer receiving carbon ion radiotherapy. According to results reported in the literature, the 12 month progression-free survival rate for patients treated with conventional radiotherapy is estimated to be 0.60. Thus, the confirmatory analysis of the primary endpoint assesses the following test problem: *H*_0_: *π *≤ 0.60 = *π*_0 _versus *H*_1_: *π *> 0.60.

The sample size calculation for the confirmatory analysis of the Phase II part of the study refers to the test problem given above that will be assessed applying a one-sided binomial test at an overall type I error rate of *α *= 0.05. A power of 1-*β *= 0.80 is aspired for the alternative of a 12 month progression-free survival rate of *π*_1 _= 0.80, i.e., for an absolute improvement of 0.20 as compared to conventional radiotherapy. As this Phase II part of PANDORA-01 is the first study providing data on the efficiency of carbon ion therapy for the treatment of recurrent rectal cancer, there is considerable uncertainty with respect to the actual 12 month progression-free survival rate resulting from carbon ion therapy in this patient population: The rate may be higher than 0.80 thus requiring fewer patients for the study; or it may be slightly lower than 0.80 while the improvement may still be clinically relevant, but with the consequence for the study that more patients than initially planned would be required to assure the desired power. For this reason, an adaptive version of Simon's optimal two-stage design is used for this Phase II study part [23,24]. This design enables to change the initially specified sample size based on the results of a planned interim analysis. If the sample size is not modified, a maximum total of 39 patients is included, with an interim analysis after *n*_1 _= 14 patients and further *n*_1 _= 25 patients recruited for the second stage if the study is continued after stage 1. Using the decision rules given, this design assures the desired power 1- *β *= 0.80 for the alternative *π*_1 _= 0.80 and has an expected sample size of 20.8 under the null hypothesis *π*_0 _= 0.60. According to the design of the Phase I part of PANDORA-01, 9 patients are already treated there with the MTD.

The primary outcome variable is the 12 month progression-free survival rate. Secondary objectives are the assessment of the 12 month overall survival rate as well as safety and tolerability.

More details on the statistical analysis will be provided in the statistical analysis plan which is finalized prior to performing any analysis and which has to be authorized by the study biostatistician and the principal investigator.

### Data safety monitoring board (DSMB)

An independent Data and Safety Monitoring Board (DSMB) will monitor the recruitment, the reported adverse events and the data quality at least twice a year. Based on its review the DSMB will provide the Principal Investigator (PI) with recommendations regarding trial modification, continuation or termination.

### Data collection and management

According to the §13 of the German GCP-Regulation all important trial documents will be archived for at least 10 years after the end of the PANDORA-01 trial. According to the §28c of the German X-Ray Regulation (RöV) and the §87 of the German Radiation Protection Regulation (StrlSchV) the informed consent forms including patients' consent for trial participation, application of irradiation and data transmission to the competent authority will be archived for at least 30 years after the end of the trial. The Study Center at the Deparment of Radiation Oncology Define will be responsible for archiving allrelevant data.

### Ethical and legal aspects

*The protocol will be condected according the guidelindes of *Good Clinical Practice (GCP) and the ethical principles described in the Declaration of Helsinki (2008 Version of the Declaration of Helsinki, adopted at the 59th WMA General Assembly, Seoul, October 2008).

The trial will be carried out by adhereing to local legal and regulatory requirements.

The study plan has obtained approval by the Institutional Review Board (IRB)/Independent Ethics Committee (EC) of the Medical Faculty Heidelberg. Before start of recruitment a positive vote of the Bundesamt für Strahlenschutz (BfS) is necessary.

## Discussion

The aim of the present PANDORA-01 trial is to evaluate the MTD for carbon ion radiotherapy in patients with recurrent rectal cancer, previously treated with radiation; subsequently, the efficacy of carbon ion radiotherapy will be evaluated in the Phase II part of the study.

To date, the possibility to treat patients with inoperable recurrent rectal cancer with radiation as a second course of radiotherapy had proven only modest effectivity, with a potential risk of treatment-related side effects [14,15,25,26]. However, the physical and biological characteristics of the carbon ion beam potentially offer a treatment alternative in this clinical situation. Previous studies from Japan on carbon ion radiotherapy for rectal cancer have shown promising results [21,27-29]. However, the published data focus on carbon ion radiotherapy applied in radiation-naïve patients. In Europe, the majority of patients presenting with recurrent rectal cancer have been treated with radiation during first-line treatment, either with preoperative chemo-radiation, short-term regimens, such as 5 × 5Gy, or with adjuvant chemoradiation, depending on tumor stage and institutional preferences.

Taking all these aspects into consideration, the present trial was designed as a Phase I/II trial firstly evaluating the MTD for carbon ion radiotherapy, followed by evaluation of efficacy in the Phase II part of the trial.

**Figure 1:**
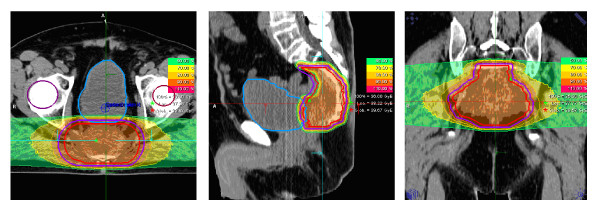
**Typical treatment plan for carbon ion radiotherapy applied with the rasterscanning technique in a patients with locally recurrent rectal cancer axial sagittal and coronal view**.

Carbon ion radiotherapy will be applied using the rasterscanning technique. A typical treatment plan can be observed in Figure 1. Patients will be followed closely to document all treatment-related side effects, as well as to assess treatment response and tumor control.

In conclusion, the PANDORA-01 study is the first trial to evaluation high-LET particle therapy with carbon ions as re-irradiation in patients with locally recurrent and unresectable rectal cancer.

## Abbreviations

ACC: Adenoid Cystic Carcinomas; BDSG: Bundesdatenschutzgesetz; CEA: Carcino-Embryonal Antigen; CHT: Chemotherapy; CRF: Case Report Form; CRP: C Reactive Protein; CT: Computer tomography; CTCAE: Common Toxicity Criteria for Adverse Events; CTV: Clinical target volume; DKFZ: Deutsches Krebsforschungszentrum; DRR: Digitally reconstructed radiograph; DVH: Dose volume histogram; GCP: Good Clinical Practice; GSI: Gesellschaft für Schwerionenforschung; GTV: Gross tumor volume; Gy: Gray; Gy E: Cobalt Gray equivalent; ICD: International Classification of Disease; LET: Linear energy transfer; LLUMC: Loma Linda University Medical Center; MGH: Massachusetts General Hospital; MR: Minor responses; MRI: Magnet resonance imaging; MTD: Maximal Tolerable Dose; n.a.: Not applicable; NIRS: National Institute of Radiological Sciences; OS: Overall survival; PR: Partial response; PTV: Planning target volume; RBE: Relative biological effectiveness; SD: Stable disease; RT: Radiation therapy; SAE: Severe Adverse Events; TME: Total Mesorectal Excision.

## Competing interests

The authors declare that they have no competing interests.

## Authors' contributions

SEC, MK, RP, REC, MD, PF and JD have developed the study concept. SEC, JD, MK wrote the study protocol and obtained ethics approval. SEC, DH, JD, JW, MWB and DJ will provide patient care. OJ will perform treatment planning and beam application for carbon ion radiotherapy. SEC, DH, JD, and MK will implement the protocol and oversee collection of the data. All authors contributed to and approved the final manuscript.

## Pre-publication history

The pre-publication history for this paper can be accessed here:

http://www.biomedcentral.com/1471-2407/12/137/prepub
